# Prognostic Value of the Acute-to-Chronic Glycemic Ratio at Admission in Heart Failure: A Prospective Study

**DOI:** 10.3390/jcm11010006

**Published:** 2021-12-21

**Authors:** Mª José Carrera, Pedro Moliner, Gemma Llauradó, Cristina Enjuanes, Laura Conangla, Juan-José Chillarón, Silvia Ballesta, Elisenda Climent, Josep Comín-Colet, Juana-Antonia Flores-Le Roux

**Affiliations:** 1 Department of Endocrinology and Nutrition, Hospital del Mar, 08003 Barcelona, Spain; jcarrera@psmar.cat (M.J.C.); jchillaron@parcdesalutmar.cat (J.-J.C.); silvia.ballesta@gmail.com (S.B.); elisenda.climent.biescas@gmail.com (E.C.); JAFlores@parcdesalutmar.cat (J.-A.F.-L.R.); 2 Department of Medicine, School of Medicine, Autonomous University of Barcelona (UAB), 08193 Cerdanyola del Vallés, Spain; 3 Department of Cardiology, Bellvitge University Hospital, L’Hospitalet de Llobregat, 08907 Barcelona, Spain; pmolinerborja@gmail.com (P.M.); cristinaenjuanes@gmail.com (C.E.); josepcomin@gmail.com (J.C.-C.); 4 BIOHEART-Cardiovascular Diseases Group, Cardiovascular, Respiratory and Systemic Diseases and Cellular Aging Program, Institut d’Investigació Biomèdica de Bellvitge—IDIBELL, L’Hospitalet del Llobregat, 08908 Barcelona, Spain; 5 Department of Clinical Sciences, School of Medicine, University of Barcelona, 08007 Barcelona, Spain; 6 Hospital de Mar Medical Research Institute—IMIM, 08003 Barcelona, Spain; 7 Centro de Investigación Biomédica en Red de Diabetes y Enfermedades Metabólicas Asociadas (CIBERDEM), Instituto de Salud Carlos III, 08209 Madrid, Spain; 8 Primary Care Center Badalona, Service Barcelonès Nord i Maresme, Catalan Health Institute, 08911 Catalonia, Spain; laura.conangla@gmail.com

**Keywords:** heart failure, diabetes mellitus, chronic complications

## Abstract

Acute hyperglycemia has been associated with worse prognosis in patients hospitalized for heart failure (HF). Nevertheless, studies evaluating the impact of glycemic control on long-term prognosis have shown conflicting results. Our aim was to assess the relationship between acute-to-chronic (A/C) glycemic ratio and 4-year mortality in a cohort of subjects hospitalized for acute HF. A total of 1062 subjects were consecutively included. We measured glycaemia at admission and estimated average chronic glucose levels and the A/C glycemic ratio were calculated. Subjects were stratified into groups according to the A/C glycemic ratio tertiles. The primary endpoint was 4-year mortality. Subjects with diabetes had higher risk for mortality compared to those without (HR 1.35 [95% CI: 1.10–1.65]; *p* = 0.004). A U-shape curve association was found between glucose at admission and mortality, with a HR of 1.60 [95% CI: 1.22–2.11]; *p* = 0.001, and a HR of 1.29 [95% CI: 0.97–1.70]; *p* = 0.078 for the first and the third tertile, respectively, in subjects with diabetes. Additionally, the A/C glycemic ratio was negatively associated with mortality (HR 0.76 [95% CI: 0.58–0.99]; *p* = 0.046 and HR 0.68 [95% CI: 0.52–0.89]; *p* = 0.005 for the second and third tertile, respectively). In multivariable analysis, the A/C glycemic ratio remained an independent predictor. In conclusion, in subjects hospitalized for acute HF, the A/C glycemic ratio is significantly associated with mortality, improving the ability to predict mortality compared with glucose levels at admission or average chronic glucose concentrations, especially in subjects with diabetes.

## 1. Introduction

The prevalence of Type 2 diabetes has quadrupled in the past three decades and is expected to continue increasing. About 1 in 11 adults worldwide now have diabetes mellitus, 90% of whom have Type 2 diabetes [1]. In Spain, according to the Di@bet.es study, its prevalence is 13.8% in the adult population (although it is undiagnosed in 6%), and the incidence of Type 2 diabetes in the adult population is 11.58 cases per 1000 persons per year [2,3].

Subjects with Type 2 diabetes have risks of death and cardiovascular events that are two to four times as great as the risks in the general population [4]. Heart failure (HF) is one of the major associated cardiovascular complications. The Framingham Study reported a two-fold increase in frequency of HF in men and a five-fold increase in women, independent of coronary artery disease and hypertension [5]. In addition, it has been reported that Type 2 diabetes increases the risk of hospitalization for HF [6]. Also, the prevalence of Type 2 diabetes seems to be increased in subjects with HF. While 10% to 15% of the general population has diabetes, a recent study suggested that 44% of subjects hospitalized for HF have Type 2 diabetes [7]. The population with both Type 2 diabetes and HF is currently between 0.3 and 0.5% of the total and is growing rapidly [8], being associated with an increased risk of cardiovascular death [7].

In recent years, it has become apparent that new glucose-lowering therapies have important HF-specific treatment risk and benefits, but those effects are not completely mediated by the improvement in glycemic control. In that sense, the potential role of glycemic control is still a matter of debate [9]. Acute hyperglycemia has been recognized as an independent determinant of adverse short-term prognosis, both in subjects with or without diabetes hospitalized for HF [9,10,11]. Nevertheless, studies evaluating the impact of glycemic control on long-term prognosis have shown conflicting results [12,13,14]. In addition, it is unknown whether hypoglycemia translates to outcomes in subjects with HF. Finally, some authors have suggested that the combination of both acute and chronic (estimated by HbA1c) glycemic value assessment may be a better prognostic predictor than glycemic value at admission or diabetes status alone [15].

Thus, given the increasing prevalence of Type 2 diabetes and HF, we need a better understanding of the independent prognostic impact of glycemic control in survival outcomes of subjects with HF. With this purpose, the aim of this study was to assess the relationship between the acute to chronic (A/C) glucose ratio and 4-year mortality in a cohort of subjects hospitalized for acute HF.

## 2. Materials and Methods

### 2.1. Study Subjects

This was a prospective, observational study. All consecutive adult subjects with acute HF admitted to the Department of Cardiology of Hospital del Mar (Barcelona, Spain) between 25 January 2005 and 29 December 2012 were enrolled. The study protocol was approved by our hospital ethics committee (Parc de Salut Mar Clinical Research Ethics Committee; approval code nº 2004/1788/I; approval date: 28 June 2004) and conducted in accordance with the Declaration of Helsinki. All subjects gave their written informed consent before participating in the study.

### 2.2. Study Protocol

The following information was recorded using a predefined standardized form: sex, age, diabetes duration, body mass index (BMI), waist, pre-admission NYHA functional class, systolic and diastolic blood pressure (SBP and DBP, respectively), cigarette smoking, alcohol intake, the use of any other medical treatment, HbA1c, lipid profile (total cholesterol, LDL-cholesterol, HDL-cholesterol, and triglycerides), serum creatinine, hemoglobin, N-terminal pro brain-type natriuretic peptide (NT-proBNP), and albumin. Hypertension was defined as systolic blood pressure >140 mmHg and/or diastolic blood pressure >90 mmHg or current treatment with antihypertensive agents [16]. The estimated glomerular filtration rate (eGFR) was estimated by the four-variable Modification of Diet in Renal Disease (MDRD) study equation. Conventional transthoracic echocardiography was used to measure left ventricular ejection fraction (LVEF).

Acute HF was diagnosed according to the current heart failure guidelines of the European Society of Cardiology (rapid or gradual onset of symptoms and/or signs of HF, severe enough for the patient to seek urgent medical attention leading to an unplanned hospital admission or an emergency department visit) [17]. Natriuretic peptides were also measured to support the diagnosis of heart failure. We excluded patients with in-hospital death, cardiogenic shock, or final diagnosis of an acute coronary syndrome.

Previously known diabetes was defined as self-reported physician-diagnosed diabetes, or use of hypoglycemic medications (insulin or oral agents). In the absence of previously known diabetes, the diagnosis of undiagnosed diabetes was based on hemoglobin A1c (HbA1c) level ≥6.5% (≥48 mmol/mol) according to American Diabetes Association criteria [17]. The diagnosis of pre-diabetes was established by HbA1c ≥5.7% and <6.5%. We were unable to distinguish between Type 1 and Type 2 diabetes, although the vast majority of our subjects with diabetes were likely to have Type 2 diabetes. 

Acute hyperglycemia was defined as a blood glucose at admission >180 mg/dL (10 mmol/L) [13]. Acute hypoglycemia was defined as blood glucose at admission <70 mg/dL (3.9 mmol/L). Average chronic glucose levels were estimated by HbA1c and expressed as percentage value, according to the following validated formula [18]:

Estimated chronic glucose levels (mg/dL) = (28.7 x HbA1c%) − 46.7

The acute/chronic (A/C) glycemic ratio was calculated in all subjects with measurements of blood glucose at admission and the estimated chronic glucose levels [15]. 

### 2.3. Outcome Measures

The primary end point of the study was 4-year all-cause mortality. Information on vital status was obtained by review of hospital and primary care medical records. 

### 2.4. Statistical Analysis

All data were tested for normality using the Shapiro–Wilk test. Data are presented as percentage mean (SD) for normally distributed quantitative variables or percentage median (interquartile range) for non-normally distributed quantitative variables. Non-normally distributed quantitative variables were used after performing a log10 transformation. One-way analysis of variance (ANOVA), or the Kruskal–Wallis test, was used for comparisons between groups of normally and non-normally distributed quantitative variables as needed. Non-normally distributed quantitative variables were used after performing a log10 transformation.The Bonferroni procedure (parametric) and the Dunn’s test (non-parametric) were used for post-hoc analyses for multiple comparisons. The association between the primary endpoint and glucose levels at admission, the estimated chronic glucose levels, the A/C glycemic ratio, and HbA1c tertiles were assessed. Survival univariate analysis was estimated with the Kaplan–Meier method and differences were tested with a long rank test. A Cox regression analysis was performed to estimate the 4-year all-cause mortality. Variables for Cox multivariate regression models were selected based on univariate analyses and on the basis of their biological plausibility based on previous literature. Two-tailed *p*-values < 0.05 were considered statistically significant. The calculations and figures were made using STATA v.13.1 for Mac (StataCorp LP, College Station, TX, USA) and GraphPad software (Prism, London, UK).

## 3. Results

### 3.1. Baseline Characteristics

In total, 1062 consecutive subjects with acute HF were included in the study (56.7% male, age: 72.6 ± 11.2 years). Of these, 497 (46.8%) had previously known diabetes, 93 (8.8%) subjects had unknown diabetes, 194 (18.2%) pre-diabetes, and 278 (26.2%) had normal glucose metabolism. Regarding diabetes treatment prior to admission in those subjects with previously known diabetes, 97 (19.5%) received dietary advice only, 200 (40.2%) were using oral hypoglycemic agents only, 56 (11.3%) were using oral hypoglycemic agents, and insulin, and 144 (29.0%) were on insulin treatment alone. 

The baseline clinical characteristics of the subjects included in the study are shown in Table 1. Subjects with diabetes were older and had higher prevalence of arterial hypertension, dyslipidemia, and peripheral artery disease compared with those without diabetes. They also had higher weight, BMI, waist circumference, higher levels of systolic blood pressure, higher concentrations of triglycerides, creatinine, and NT-proBNP, but lower levels of total-, LDL-, and HDL-cholesterol (in the context of higher percentage of subjects under lipid-lowering treatments) and hemoglobin. Moreover, subjects with diabetes had a higher prevalence of chronic kidney disease, heart failure of ischemic etiology, and NYHA class III–IV. There were no differences between groups for gender distribution, smoking habit, and percentage of subjects with LVEF ≤ 40 %. The use of angiotensin-converting enzyme inhibitors was lower in subjects with diabetes, while the use of angiotensin receptor blockers, aldosterone antagonists, diuretics, and amiodarone was similar in both groups. Statin, nitrates, and antiplatelet drugs were used more commonly in subjects with diabetes mellitus. Subjects with diabetes had higher ratios of acute heart failure hospitalization during the last year.

**Figure 1:**
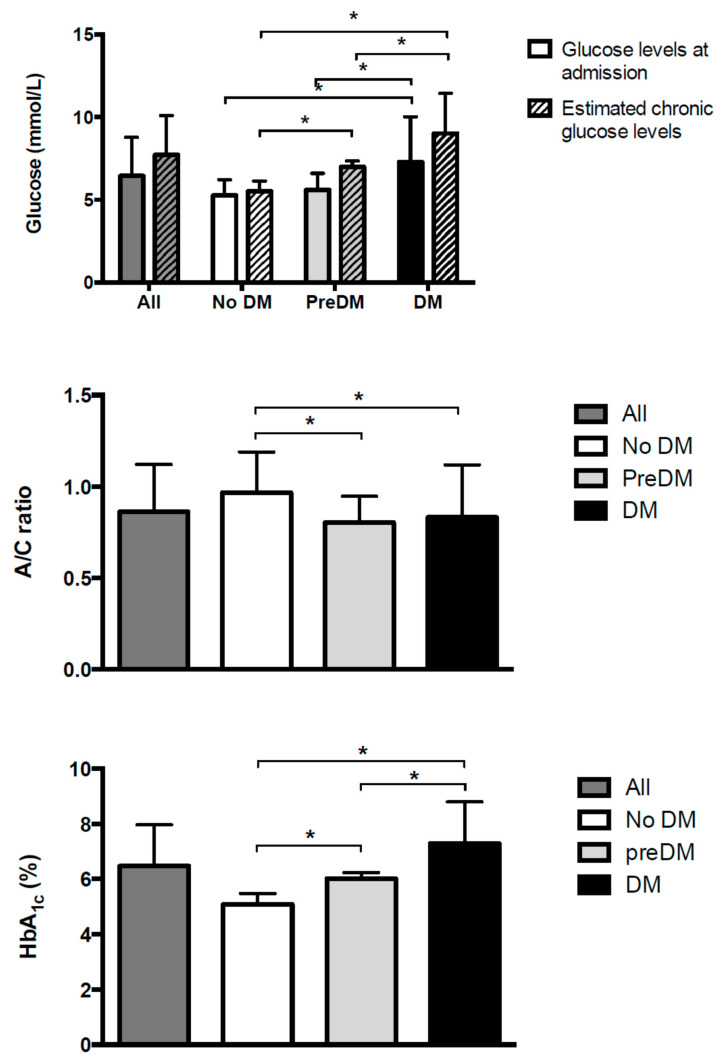
Glucose at admission and estimated chronic glucose levels (top panel), acute to chronic glycemic ratio (mid panel), and HbA1c (bottom panel) for subjects admitted to the hospital for acute heart failure (whole population and stratified by diabetes status) * *p* < 0.005.

Subjects with diabetes (diagnosed and undiagnosed), when compared with those with pre-diabetes and without diabetes, had higher glucose levels at admission (7.3 ± 2.7 mmol/L vs. 5.6 ± 1.0 mmol/L vs. 5.3 ± 1.0 mmol/L; *p* for trend <0.001) (Figure 1, top panel), higher estimated chronic glucose levels (9.0 ± 2.4 mmol/L vs. 7.0 ± 0.4 mmol/L vs. 5.5 ± 0.6 mmol/L; *p* for trend < 0.001) (Figure 1, top panel), lower A/C glycemic ratio (0.83 ± 0.29 vs. 0.80 ± 0.14 vs. 0.97 ± 0.22; *p* for trend <0.001) (Figure 1, middle panel), and higher values of HbA1c (7.3 ± 1.5% (56.0 ± 16.4 mmol/mol) vs. 6.0 ± 0.2% (42.0 ± 2.2 mmol/mol) vs. 5.1 ± 0.4% (32.0 ± 4.4 mmol/mol); *p* for trend <0.001) (Figure 1, bottom panel) compared to those subjects with pre-diabetes and without diabetes.

### 3.2. Mortality during the Follow-Up

**Figure 2:**
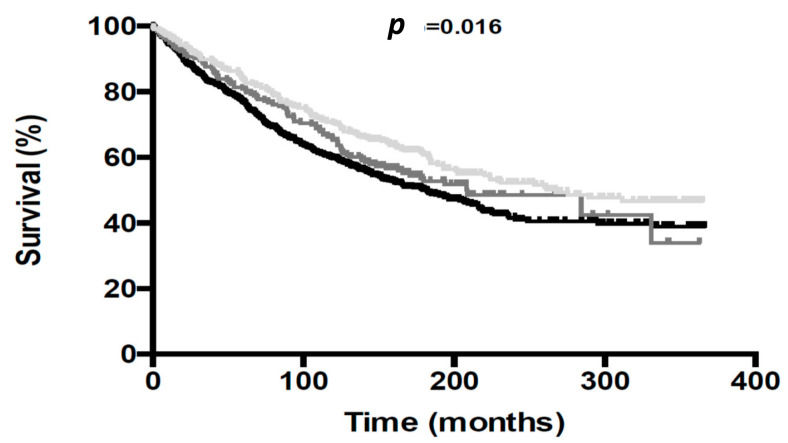
Kaplan–Meier survival curves for all-cause mortality according to glycemic category: normal glucose metabolism (grey line), pre-diabetes (dark grey line) and diabetes (black line).

Over the 4-year follow-up period, there were 546 deaths (51.4%), 319 (58.4%) subjects with diabetes, 94 (17.2%) subjects with pre-diabetes, and 133 (24.4%) subjects without diabetes. The Kaplan–Meier survival curves are shown in Figure 2. Subjects with diabetes had higher risk for 4-year mortality compared to those without diabetes (HR 1.35 [95% CI: 1.10–1.65]; *p* = 0.004). On the contrary, subjects with pre-diabetes did not show higher mortality rates compared to those without diabetes (HR 1.19 [95% CI: 0.91–1.56], *p* = 0.201). 

**Figure 3:**
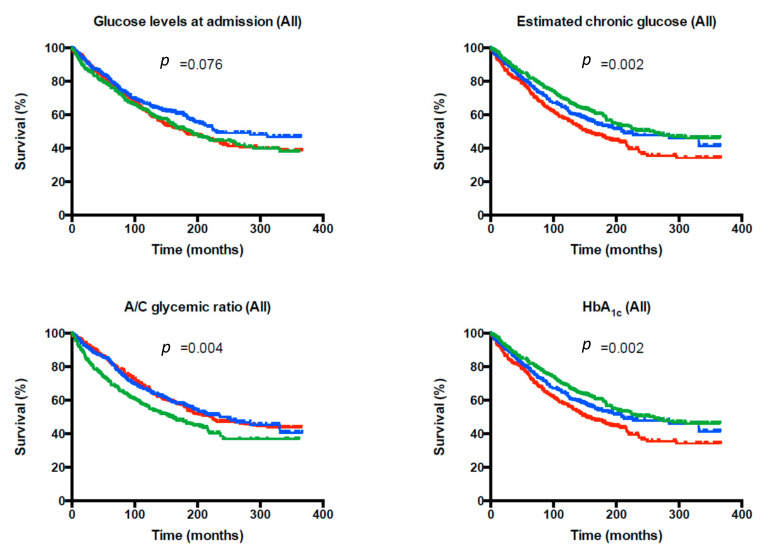
Kaplan–Meier survival curves for all-cause mortality according to the tertiles (first tertile: green, second tertile: blue, third tertile: red) of glucose levels at admission, estimated chronic glucose levels, acute to chronic glycemic ratio, and HbA1c for the whole population.

**Figure 4:**
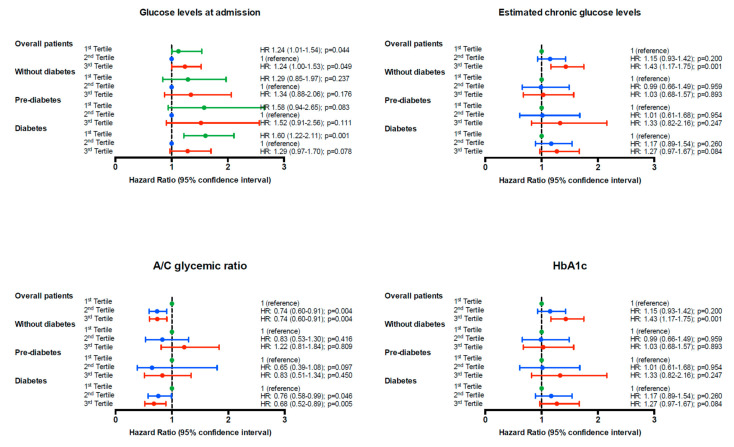
Unadjusted hazard ratio (95% CI) of the end point (4-year mortality for all-cause) grouped according to tertiles (first tertile: green, second tertile: blue, third tertile: red) of glucose levels at admission, estimated chronic glucose levels, the acute to chronic glycemic ratio, and HbA1c in the whole population and stratified according to glucose status.

Figure 3 shows the Kaplan–Meier survival curves for all-cause mortality according to tertiles of glucose levels at admission, estimated chronic glucose levels, A/C glycemic ratio, and HbA1c for the whole population. Similar results were obtained when Cox models were built for the primary end-point. Figure 4 shows the unadjusted hazard ratio (HR) and the 95% confidence interval (95% CI) for glucose levels at admission, estimated chronic glucose levels, the A/C glycemic ratio, and HbA1c tertiles in the whole population, stratified according to glucose status. While in subjects without diabetes and subjects with pre-diabetes, no association was found with 4-year mortality, the results found in the whole population were particularly evident in subjects with diabetes. In this subgroup, a U-shape curve association was found between glucose levels at admission and the 4-year mortality risk. The first tertile was significantly associated with higher 4-year mortality (HR 1.60 [95% CI: 1.22–2.11]; *p* = 0.001) and the third tertile showed a trend towards higher mortality (HR 1.29 [95% CI: 0.97–1.70]; *p* = 0.078)). In addition, the A/C glycemic ratio was negatively associated with 4-year mortality in subjects hospitalized for acute HF (HR 0.76 [95% CI: 0.58–0.99]; *p* = 0.046 and HR 0.68 [95% CI: 0.52–0.89]; *p* = 0.005 for the second and the third tertile, respectively). Acute hypoglycemia was associated with a 4-year mortality risk in this population (HR 2.84 [95% CI: 1.55–5.19]; *p* = 0.001).

To further identify the potential independent predictors associated with all-cause mortality and to evaluate the potential role of both acute and chronic glycemic control, multivariate Cox regression analyses were performed. Candidate variables were selected based on univariate analyses and on the basis of previously described risk factors in subjects with heart failure. In the univariate analysis, age, arterial hypertension, chronic kidney disease, troponin T, NT-proBNP, III-IV functional class of the NYHA, heart failure of ischemic etiology, previous peripheral artery disease, previous chronic obstructive pulmonary disease, and a previous history of heart failure admission during the last year were positively associated with 4-year mortality (Table 2). BMI, hemoglobin, and serum albumin concentrations were negatively associated with all-cause mortality. Table 3 shows the results after adjusting for multiple risk factors regarding the association between diabetes status and the risk of 4-year mortality. Subjects in the third tertile of A/C glycemic ratio (mean glucose at admission: 7.7 mmol/L (range 4.3–27.0); mean HbA1c: 5.5% (range 5.1–7.5) (37 mmol/mol; 32–58 mmol/mol)), which represents those patients with a good chronic glycemic control, but acute stress hyperglycemia at admission, had a lower risk of 4-year mortality (adjusted HR 0.79 [95% CI: 0.64–0.99]; *p* = 0.040) compared to those in the first tertile (mean glucose at admission: 5.5 mmol/L (range 1.1–14.7); mean HbA1c: 7.3% (range 4.7–15.2) (56 mmol/mol; range 28–143). These results would suggest that acute hypoglycemia at admission, or at least relative hypoglycemia (i.e., lower levels than the expected for chronic glycemic control), is deleterious in subjects with diabetes. Other variables that were independently associated with 4-year mortality were age, III-IV functional class of the NYHA, heart failure of ischemic etiology, previous peripheral artery disease, and NT-proBNP concentrations. When the results were analyzed stratifying the subjects according to the etiology of heart failure (non-ischemic vs. ischemic), the association between the third tertile of A/C glycemic ratio and the 4-year mortality was still significant for the ischemic group (unadjusted HR 0.71 [95% CI: 0.53–0.95]; *p* = 0.023 and adjusted HR 0.69 [95% CI: 0.49–0.97]; *p* = 0.034). Similar results were found when HbA1c (or estimated chronic glucose levels) was used instead of A/C glycemic ratio, with a higher risk of 4-year mortality for those subjects in the third tertile (adjusted HR 1.26 [95% CI: 1.02–1.57], *p* = 0.032). No association was found between 4-year mortality and glucose status, acute hypo- or hyperglycemia, or glucose levels at admission.

## 4. Discussion

The main finding of the present study is that the A/C glycemic ratio was significantly associated with 4-year mortality in subjects hospitalized for acute HF, improving the ability to predict the 4-year mortality compared with glucose levels at admission or the average chronic glucose concentrations. These results were particularly evident in subjects with diabetes, in whom a high glucose level at admission is not always an index of stress hyperglycemia; furthermore, lower glucose concentrations or an intensive glycemic control can be sometimes detrimental. Therefore, the assessment of the A/C glycemic ratio could better identify true stress hyperglycemia rather than the glucose levels at admission alone, taking into account at the same time relative hypoglycemia and thus improving the discrimination of subjects at high risk.

Acute hyperglycemia has been recognized as an independent determinant of adverse short-term prognosis, both in subjects with or without diabetes hospitalized for HF (9–11). Nevertheless, studies evaluating the impact of glycemic control on long-term prognosis have shown conflicting results (12–14). To the best of our knowledge, this is the first study evaluating the potential role of the A/C glycemic ratio as a prognostic factor in subjects hospitalized for acute HF. In our study and accordingly to previous results [19], subjects with diabetes had a higher risk for 4-year mortality compared with subjects without diabetes or pre-diabetes. In addition, we found that the A/C glycemic ratio was significantly associated with 4-year mortality in the whole population and particularly in subjects with diabetes and in subjects with heart failure of ischemic etiology. While the average chronic glucose level increased in parallel with the increase of 4-year mortality in the whole population (showing a trend towards significance in subjects with diabetes), the glucose levels at admission presented a U-shape curve association. This later finding suggests that both acute hyper- and hypoglycemia could have a negative impact on mortality in subjects with diabetes hospitalized for acute HF. However, the A/C glycemic ratio, which integrates both acute and chronic glycemic control, was inversely associated with 4-year mortality, particularly in subjects with diabetes. These results might suggest that an imbalance between glycaemia at admission and chronic metabolic control can be detrimental (especially for those with glucose levels at admission lower than the expected for chronic glycemic control), resulting in higher mortality rates and adding a better prognostic value than each component alone. 

In that sense, Fujino et al. showed that acute hyperglycemia had worse in-hospital outcome in subjects with AMI. However, in subjects with glycemia at admission ≥200 mg/dL, the in-hospital mortality rate was lower in those with HbA1c ≥ 6.5% compared with those subjects with HbA1c < 6.5% (5.5 vs. 18.9%; *p* = 0.010). [20]. Marenzi et al. demonstrated that the A/C glycemic ratio was associated with in-hospital mortality, morbidity, and infarct size in subjects with acute myocardial infarction (AMI), improving the prediction capacity of glycaemia at admission and HbA1c, with the results particularly evident in subjects with diabetes [15]. Finally, in COVID-19 patients, an association between worst outcomes (in-hospital mortality, ICU admission, and mechanical ventilation) and the third A/C glycemic ratio tertile was found (adjusted HR 3.96 [95% CI 1.35–11.59], *p* = 0.012). Moreover, an increased mortality rate was demonstrated in the first and third A/C glycemic ratio tertiles, with glycemic levels at admission 18% under and 22% above chronic levels, respectively [21].

Hypoglycemia has been associated with an increased risk of cardiovascular events and mortality [22,23]. This association is likely to be multifactorial [24]. Three randomized clinical trials comparing the effect of more versus less intensive glycemic control among individuals with established Type 2 diabetes at increased cardiovascular risk (ACCORD, ADVANCE, and VADT) did not show significant reductions in cardiovascular events or mortality [25,26,27,28]. In fact, mortality increased in the ACORD trial and all three studies showed a significant association between severe hypoglycemia and mortality. Whether the association was causal or because of confounding factors remains unclear. Nevertheless, the results of our study take a step further, suggesting that lower levels than the expected for chronic glycemic control are also associated with mortality. Hypoglycemic events trigger a contra-regulatory response that involves multiple stress pathways and sympathetic nervous system activation. These systemic changes have substantial hemodynamic, pro-inflammatory, and pro-atherothrombotic effects that worsen cardiac function [24]. Moreover, it is well-known that up to 25 percent of critical patients have a partial ACTH deficiency that would condition a relative suprarenal insufficiency [29]. This fact might explain the increased mortality without a stress hyperglycemia response.

HF is common in Type 2 diabetes and vice versa, leading to a mutual negative impact on prognosis [30]. While the prevalence of Type 2 diabetes in general population is 10–15% [2], prevalence of Type 2 diabetes in subjects with HF is 25–30%, a proportion that increases to 40% in those subjects hospitalized for HF [7,31]. In that sense, our results are in accordance with previous data and confirm that in subjects with HF at high-risk for hospitalization, the prevalence of diabetes in subjects hospitalized for acute HF was higher (55.6%) and increased to 74% when pre-diabetes is also taken into account. Moreover, subjects with diabetes had a worse clinical profile compared with subjects without diabetes (with higher numbers of comorbidities, higher decline of renal function, higher concentrations of NT-proBNP, worst NYHA class, and higher rates of hospital admission for acute HF in the previous year), which probably justifies the higher 4-year mortality rates. Nevertheless, the association between the A/C glycemic ratio and 4-year mortality remained significant after adjusting for the other potential prognosis factors, which reinforces our results. Thus, all these results highlight the importance of diabetes and its metabolic control in the progression and prognosis of HF. 

Knowledge about the complex interplay between Type 2 diabetes and HF has dramatically changed recently due to appearance of new glucose-lowering drugs. Recently performed cardiovascular outcome trials (CVOTs) have shown that many glucose-lowering drugs may variably influence the risk or progression of HF. In that sense, the sodium-glucose co-transporter 2 inhibitors (iSGLT-2) have demonstrated a risk reduction of hospitalization for HF (HR between 0.61–0.73) and the risk reduction of MACE (cardiovascular death, myocardial infarction, and ischemic stroke) in the HF subgroup (DECLARE TIMI 58, CANVAS, EMPA-REG OUTCOME, CREDENCE) [32,33,34,35]. The interplay between both diseases is complex and multifactorial, and the mechanisms of potential benefits in HF are not fully elucidated, but may extend beyond glycemic control. iSGLT-2 have shown reductions in HbA1c between 0.48–1.16% compared with placebo, a slight improvement to justify a 27–39% reduction of hospitalization for HF. Given their potential benefits in the population with HF, iSGLT-2 are currently being investigated in the treatment of subjects with chronic HF. In that sense, the DAPA-HF demonstrated a risk reduction of 26% of the primary composite endpoint (mortality or worsening NYHA class) and a risk reduction of 25% for the secondary composite endpoint (mortality or hospitalization for HF) [36]. Furthermore, the EMPEROR-Reduced trial found that the combined risk of cardiovascular death or hospitalization for heart failure was 25% lower among patients who received empagliflozin, regardless of the presence or absence of diabetes [37]. This recent evidence may lead to a misperception that metabolic control is not important in subjects with diabetes and HF. However, our study, designed prior to the implementation of these new glucose-lowering drugs, highlights the importance of the potential role of glucose changes in cardiovascular outcomes.

The strengths of the current study include its large sample size, the prospective design, a well-characterized population, adjustment for a variety of risk factors, and a long-term follow-up. Nevertheless, our study is not exempt of limitations. Firstly, our cohort includes subjects hospitalized for acute HF; thus our results cannot be extrapolated to the whole HF population. Secondly, the identification of diabetes subtypes was not feasible, although other diabetes subtypes different than Type 2 diabetes may represent a lower proportion. Thirdly, information on cause of death or hospital readmission was not collected. Finally, HbA1c values, which might have had an impact on HF prognosis, were not recorded during the follow-up.

## 5. Conclusions

In conclusion, we have demonstrated that the A/C glycemic ratio in subjects with diabetes hospitalized for acute HF is closely associated with 4-year mortality. Use of the A/C glycemic ratio may be particularly valuable in subjects with diabetes with poor chronic glycemic control because it may identify true stress hyperglycemia, which has been associated with worse prognosis.

## Figures and Tables

**Table 1:** Baseline characteristics of the whole cohort of patients with acute heart failure stratified by diabetes status.

	All (*n* = 1062)	Patients without Diabetes (*n* = 278)	Patients with Pre-Diabetes (*n* = 194)	Patients with Diabetes (*n* = 590)	*p* for Trend
Male sex (*n*, %)	602 (56.7)	153 (55.0)	112 (57.7)	337 (57.1)	0.803
Age (years)	72.6 ± 11.2	71.2 ± 12.6	72.6 ± 13.2	73.2 ± 9.7 ^¤^	0.046
Current smokers (*n*, %)	157 (14.8)	39 (14.0)	34 (17.5)	84 (14.2)	0.786
BMI (kg/m^2^)	28.4 ± 5.9	27.2 ± 5.3	28 ± 6.5	29.1 ± 5.8 ^¤ ¥^	<0.001
Waist (cm)	103.4 ± 14.2	98.9 ± 14.4	101.7 ± 13.8	105.9 ± 14.2 ^¤ ¥^	<0.001
Hypertension (*n*, %)	862 (81.2)	191 (68.7)	153 (78.9)	518 (87.8) * ^¤ ¥^	<0.001
Systolic blood pressure (mmHg)	123.5 ± 22.0	120.1 ± 21.6	120.0 ± 22.2	126.1 ± 21.7 ^¤ ¥^	<0.001
Diastolic blood pressure (mmHg)	67.2 ± 12.9	67.3 ± 13.2	66.6 ± 12.6	67.3 ± 12.8	0.768
Dyslipidemia (*n*, %)	593 (55.8)	109 (39.2)	81 (41.8)	403 (68.3) ^¤ ¥^	<0.001
Statin use (*n*, %)	633 (59.6)	124 (44.6)	101 (52.1)	408 (69.2) * ^¤ ¥^	<0.001
Total cholesterol (mmol/L)	3.92 ± 0.99	4.14 ± 1.04	4.08 ± 0.97	3.77 ± 1.03 ^¤ ¥^	<0.001
LDL-C (mmol/L)	2.22 ± 0.80	2.43 ± 0.81	1.55 ± 0.78	2.08 ± 0.78 ^¤ ¥^	<0.001
HDL-C (mmol/L)	1.10 ± 0.32	1.13 ± 0.33	1.17 ± 0.28	1.07 ± 0.33 ^¥^	<0.001
Triglycerides (mmol/L)	1.40 ± 0.66	1.40 ± 0.74	1.30 ± 0.54	1.44 ± 0.66 ^¥^	0.049
COPD (*n*, %)	237 (22.3)	51 (18.3)	44 (22.7)	142 (24.1)	0.152
Previous stroke (*n*, %)	119 (11.2)	28 (10.1)	18 (9.2)	73 (12.4)	0.379
Peripheral vasculopathy (*n*, %)	176 (16.6)	33 (11.9)	21 (10.8)	122 (20.7) ^¤ ¥^	<0.001
**Laboratory values**					
Creatinine (mmol/L)	0.12 ± 0.06	0.12 ± 0.05	0.011 ± 0.05	0.12 ± 0.07 ^¥^	0.004
eGFR MDRD < 60 (mL/min/1.73m^2^)	277 (26.1)	61 (21.9)	35 (18.0)	18 (3.1) ^¤ ¥^	<0.001
Hemoglobin (g/L)	12.6 ± 2.8	12.7 ± 1.9	12.9 ± 2.1	13.4 ± 3.3	0.047
Albumin (g/dL)	3.78 ± 0.48	3.78 ± 0.45	3.90 ± 0.51	3.76 ± 0.48 * ^¥^	0.001
NT-proBNP (pg/mL)	1626 (700–4051)	1412 (639–3425)	1537 (652–3246)	1775 (719–4456)	0.103
LVEF ≤40% (*n*, %)	485 (45.7)	144 (51.8)	81 (41.8)	260 (44.1)	0.057
NYHA class (*n*, %)					0.011^¤^
-Class l	146 (13.7)	38 (13.7)	45 (23.2)	65 (11.0)	
-Class II	440 (41.4)	128 (46.0)	70 (36.1)	240 (40.8)	
-Class III	388 (36.5)	94 (33.8)	58 (29.9)	236 (40.0)	
-Class IV	88 (8.3)	18 (6.5)	21 (10.8)	49 (8.3)	
HF etiology (*n*, %)					<0.001
-Ischemic	428 (40.3)	83 (29.9)	60 (30.9)	285 (48.3) ^¤ ¥^	
-Hypertensive	372 (35.0)	94 (33.8)	72 (37.1)	206 (34.9)	
-Others	262 (24.7)	101 (36.3)	62 (32.0)	189 (32.0)	
Atrial fibrillation (*n*, %)	354 (33.3)	100 (36.0)	74 (38.1)	180 (30.5)	0.083
Implantable cardioverter defibrillator (*n*, %)	15 (1.4)	8 (2.9)	0 (0)	7 (1.2)	0.011
Last-year acute heart failure admission (*n*, %)	858 (80.8)	212 (76.3)	149 (76.8)	497 (84.2)	0.005
**Cardiovascular medication**					
-ACE inhibitors (*n*, %)	632 (59.5)	182 (65.5)	110 (56.7)	340 (57.6)^¤^	0.029
-ARBs (*n*, %)	172 (16.2)	44 (15.8)	39 (20.1)	89 (15.1)	0.308
-**β**-Blockers (*n*, %)	927 (87.3)	251 (90.3)	161 (83.0)	515 (87.3) * ^¤^	0.047
-Nitrates (*n*, %)	327 (30.8)	54 (19.4)	41 (21.1)	233 (39.5) ^¤ ¥^	<0.001
-Aldosterone antagonists (*n*, %)	397 (37.4)	115 (41.4)	71 (36.6)	211 (35.8)	0.287
-Diuretics (*n*, %)	958 (90.2)	247 (88.8)	173 (89.2)	538 (91.2)	0.433
-Antiagregant (*n*, %)	494 (46.5)	92 (33.1)	73 (37.6)	329 (55.8) ^¤ ¥^	<0.001
-Amiodarone (*n*, %)	102 (9.6)	33 (11.9)	18 (9.3)	51 (8.6) ^¤ ¥^	0.334

Data are given as percentages, mean ± SD or median (interquartile range). BMI, body mass index; LDL-C, low-density lipoprotein cholesterol; HDL-C, high-density lipoprotein; COPD, chronic obstructive pulmonary disease; eGFR-MDRD, estimated glomerular filtration rate by the four-variable Modification of Diet in Renal Disease; NT-proBNP, N-terminal pro brain-type natriuretic peptide; LVEF, left ventricular ejection fraction; NYHA class, The New York Heart Association functional classification; HF, heart failure; ACE inhibitors, angiotensin-converting enzyme inhibitors; ARBS angiotensin II receptor blockers. * *p* < 0.05 patients without diabetes vs. patients with pre-diabetes, ^¤^ *p* < 0.05 patients without diabetes vs. patients with diabetes, ^¥^ *p* < 0.05 patients with pre-diabetes vs. patients with diabetes.

**Table 2:** Univariate Cox regression analysis of 4-year all-cause death.

Variables	HR (95% CI)	*p* Value
Age (years)	1.04 (1.03–1.05)	<0.001
Sex (female vs. male)	1.04 (0.88–1.23)	0.637
Smoking (yes vs. no)	0.97 (0.89–1.05)	0.421
BMI (kg/m^2^)	0.97 (0.96–0.99)	<0.001
Hypertension (yes vs. no)	1.36 (1.08–1.71)	<0.010
Dyslipidemia (yes vs. no)	1.01 (0.85–1.20)	0.884
Statin use (yes vs. no)	1.06 (0.89–1.26)	0.532
CKD (yes vs. no)	1.68 (1.41–2.02)	<0.001
Hemoglobin (g/dL)	0.93 (0.89–0.97)	<0.001
Albumin (g/dL)	0.56 (0.47–0.68)	<0.001
Troponin T	1.14 (1.06–1.24)	0.001
NT-proBNP (pg/mL)	2.31 (1.98–2.70)	<0.001
LVEF ≤ 40% (yes vs. no)	0.93 (0.79–1.11)	0.431
NYHA class (III–IV vs. I–II)	1.79 (1.50–2.13)	<0.001
HF of ischemic etiology (yes vs. no)	1.49 (1.26–1.77)	<0.001
Previous stroke (yes vs. no)	1.29 (0.99–1.65)	0.050
Previous peripheral artery disease (yes vs. no)	1.42 (1.15–1.75)	0.001
Previous COPD (yes vs. no)	1.34 (1.11–1.63)	0.003
Last-year acute heart failure admission (yes vs. no)	1.88 (1.46–2.42)	<0.001

HR: hazard ratio; CI: confidence interval; BMI: body mass index; CKD: chronic kidney disease; ProBNP: N-terminal pro-brain-type natriuretic peptide; HF: heart failure; NYHA: New York Heart Association; COPD: chronic obstructive pulmonary disease.

**Table 3:** Multivariate Cox regression analysis of 4-year all-cause death in the whole population of patients with acute heart failure.

Variables	HR (95% CI)	*p* Value
Age (years)	1.03 (1.02–1.04)	<0.001
HF of ischemic etiology (yes vs. no)	1.38 (1.12–1.71)	0.003
NT-proBNP (pg/mL)	2.05 (1.68–2.51)	<0.001
A/C glycemic ratio (third tertile)	0.79 (0.64–0.99)	0.040

Variables included in the model were: Age, sex, diabetes status (without diabetes, prediabetes and diabetes) smoking habit, body mass index, arterial hypertension (yes vs. no), chronic kidney disease, hemoglobin, serum albumin, N-terminal pro-brain-type natriuretic peptide (NT-proBNP) (logarithm), III–IV functional class of the New York Heart Association, heart failure (HF) of ischemic etiology, left ventricular ejection fraction, previous peripheral artery disease, stroke, chronic obstructive pulmonary disease, and a previous history of HF admission during the last year. A/C glycemic ratio, acute/chronic glycemic ratio.

## Data Availability

J.C.-C. and J.-A.F.-L.R. are the guarantors of this work and, as such, had full access to all the data in the study and take responsibility for the integrity of the data and the accuracy of the data analysis.

## References

[1] Zheng Y., Ley S.H., Hu F.B. (2018). Global aetiology and epidemiology of type 2 diabetes mellitus and its complications. Nat. Rev. Endocrinol..

[2] Soriguer F., Goday A., Bosch-Comas A., Bordiú E., Calle-Pascual A., Carmena R., Casamitjana R., Castaño L., Castell C., Catalá M. (2012). Prevalence of diabetes mellitus and impaired glucose regulation in Spain: The Di@betes Study. Diabetologia.

[3] Rojo-Martínez G., Valdés S., Soriguer F., Vendrell J., Urrutia I., Pérez V., Ortega E., Ocón P., Montanya E., Menéndez E. (2020). Incidence of diabetes mellitus in Spain as results of the nation-wide cohort di@betes study. Sci. Rep..

[4] Rawshani A., Rawshani A., Franzén S., Eliasson B., Svensson A.-M., Miftaraj M., McGuire D.K., Sattar N., Rosengren A., Gudbjörnsdottir S. (2017). Mortality and Cardiovascular Disease in Type 1 and Type 2 Diabetes. N. Engl. J. Med..

[5] Kannel W.B., Hjortland M., Castelli W.P. (1974). Role of diabetes in congestive heart failure: The Framingham study. Am. J. Cardiol..

[6] Rawshani A., Rawshani A., Franzén S., Sattar N., Eliasson B., Svensson A.-M., Zethelius B., Miftaraj M., McGuire D.K., Rosengren A. (2018). Risk Factors, Mortality, and Cardiovascular Outcomes in Patients with Type 2 Diabetes. N. Engl. J. Med..

[7] Cavender M.A., Steg P.G., Smith S.C., Eagle K., Ohman E.M., Goto S., Kuder J., Im K., Wilson P.W., Bhatt D.L. (2015). REACH Registry Investigators. Impact of Diabetes Mellitus on Hospitalization for Heart Failure, Cardiovascular Events, and Death. Circulation.

[8] Echouffo-Tcheugui J.B., Xu H., DeVore A.D., Schulte P.J., Butler J., Yancy C.W., Bhatt D.L., Hernandez A.F., Heidenreich P.A., Fonarow G.C. (2016). Temporal trends and factors associated with diabetes mellitus among patients hospitalized with heart failure: Findings from Get With The Guidelines–Heart Failure registry. Am. Heart J..

[9] Mebazaa A., Gayat E., Lassus J., Meas T., Mueller C., Maggioni A., Peacock F., Spinar J., Harjola V.P., van Kimmenade R. (2013). Association between elevated blood glucose and outcome in acute heart failure: Results from an international observational cohort. J. Am. Coll. Cardiol..

[10] Helfand B.K.I., Maselli N.J., Lessard D.M., Yarzebski J., Gore J.M., McManus D.D., Saczynski J.S., Goldberg R.J. (2015). Elevated serum glucose levels and survival after acute heart failure: A population-based perspective. Diabetes Vasc. Dis. Res..

[11] Cho J.Y., Kim K.H., Lee S.E., Cho H.-J., Lee H.-Y., Choi J.-O., Jeon E.S., Kim M.S., Kim J.J., Hwang K.K. (2020). Admission Hyperglycemia as a Predictor of Mortality in Acute Heart Failure: Comparison between the Diabetics and Non-Diabetics. J. Clin. Med..

[12] Itzhaki Ben Zadok O., Kornowski R., Goldenberg I., Klempfner R., Toledano Y., Biton Y., Fisman E.Z., Tenenbaum A., Golovchiner G., Kadmon E. (2017). Admission blood glucose and 10-year mortality among patients with or without pre-existing diabetes mellitus hospitalized with heart failure. Cardiovasc. Diabetol..

[13] Newton J.D., Squire I.B. (2006). Glucose and haemoglobin in the assessment of prognosis after first hospitalisation for heart failure. Heart.

[14] Targher G., Dauriz M., Laroche C., Temporelli P.L., Hassanein M., Seferovic P.M.M., Drozdz J., Ferrari R., Anker S., Coats A. (2017). In-hospital and 1-year mortality associated with diabetes in patients with acute heart failure: Results from the ESC-HFA Heart Failure Long-Term Registry. Eur. J. Heart Fail..

[15] Marenzi G., Cosentino N., Milazzo V., Metrio M., De Cecere M., Mosca S., Rubino M., Campodonico J., Moltrasio M., Marana I. (2018). Prognostic value of the acute-to-chronic glycemic ratio at admission in acute myocardial infarction: A prospective study. Diabetes Care.

[16] Chobanian A.V., Bakris G.L., Black H.R., Cushman W.C., Green L.A., Izzo J.L., Jones D.W., Materson B.J., Oparil S., Wright J.T. (2003). The Seventh Report of the Joint National Committee on Prevention, Detection, Evaluation, and Treatment of High Blood Pressure: The JNC 7 Report. J. Am. Med. Assoc. JAMA.

[17] (2010). Standards of medical care in diabetes-2010. Diabetes Care.

[18] Nathan D.M., Kuenen J., Borg R., Zheng H., Schoenfeld D., Heine R.J. (2008). Translating the A1C assay into estimated average glucose values. Diabetes Care.

[19] Flores-Le Roux J.A., Comin J., Pedro-Botet J., Benaiges D., Puig-de Dou J., Chillarón J.J., Goday A., Bruguera J., Cano-Perez J.F. (2011). Seven-year mortality in heart failure patients with undiagnosed diabetes: An observational study. Cardiovasc. Diabetol..

[20] Fujino M., Ishihara M., Honda S., Kawakami S., Yamane T., Nagai T., Nakao K., Kanaya T., Kumasaka L., Asaumi Y. (2014). Impact of acute and chronic hyperglycemia on in-hospital outcomes of patients with acute myocardial infarction. Am. J. Cardiol..

[21] Ramon J., Llauradó G., Güerri R., Climent E., Ballesta S., Benaiges D., López-Montesinos I., Navarro H., Fernández N., Carrera M.J. (2021). Acute-to-Chronic Glycemic Ratio as a Predictor of COVID-19 Severity and Mortality. Diabetes Care.

[22] Lee A.K., Warren B., Lee C.J., McEvoy J.W., Matsushita K., Huang E.S., Sharrett A.R., Coresh J., Selvin E. (2018). The association of severe hypoglycemia with incident cardiovascular events and mortality in adults with type 2 diabetes. Diabetes Care.

[23] Malik A.H., Yandrapalli S., Aronow W.S., Jain D., Frishman W.H., Panza J.A., Cooper H.A. (2020). Severe Hypoglycemia and Risk of Subsequent Cardiovascular Events: Systematic Review and Meta-Analysis of Randomized Controlled Trials. Cardiol. Rev..

[24] Amiel S.A., Aschner P., Childs B., Cryer P.E., de Galan B.E., Frier B.M. (2019). International Hypoglycaemia Study Group. Hypoglycaemia, cardiovascular disease, and mortality in diabetes: Epidemiology, pathogenesis, and management. Lancet Diabetes Endocrinol..

[25] Gerstein H.C., Miller M.E., Byington R.P., Goff D.C., Bigger J.T., Buse J.B., Cushman W.C., Genuth S., Ismail-Beigi F., Zoungas S. (2008). Action to Control Cardiovascular Risk in Diabetes Study Group.Effects of Intensive Glucose Lowering in Type 2 Diabetes. N. Engl. J. Med..

[26] Patel A., MacMahon S., Chalmers J., Neal B., Billot L., Woodward M., Marre M., Cooper M., Glasziou P., ADVANCE Collaborative Group (2008). Intensive Blood Glucose Control and Vascular Outcomes in Patients with Type 2 Diabetes. N. Engl. J. Med..

[27] Agrawal L., Azad N., Bahn G.D., Ge L., Reaven P.D., Hayward R.A., Reda D.J., Emanuele N.V., VADT Study Group (2018). Long-term follow-up of intensive glycaemic control on renal outcomes in the Veterans Affairs Diabetes Trial (VADT). Diabetologia.

[28] Meier M., Hummel M. (2009). Cardiovascular disease and intensive glucose control in type 2 diabetes mellitus: Moving practice toward evidence-based strategies. Vasc. Health Risk Manag..

[29] Arafah B.M. (2006). Review: Hypothalamic pituitary adrenal function during critical illness: Limitations of current assessment methods. J. Clin. Endocrinol. Metab..

[30] Carrasco-Sánchez F.J., Gomez-Huelgas R., Formiga F., Conde-Martel A., Trullàs J.C., Bettencourt P., Arévalo-Lorido J.C., Pérez-Barquero M.M., RICA investigators (2014). Association between type-2 diabetes mellitus and post-discharge outcomes in heart failure patients: Findings from the RICA registry. Diabetes Res. Clin. Pract..

[31] Farré N., Vela E., Clèries M., Bustins M., Cainzos-Achirica M., Enjuanes C., Moliner P., Ruiz S., Verdú-Rotellar J.M., Comín-Colet J. (2017). Real world heart failure epidemiology and outcome: A population-based analysis of 88,195 patients. PLoS ONE.

[32] Wiviott S.D., Raz I., Bonaca M.P., Mosenzon O., Kato E.T., Cahn A., Silverman M.G., Zelniker T.A., Kuder J.F., Murphy S.A. (2019). Dapagliflozin and Cardiovascular Outcomes in Type 2 Diabetes. N. Engl. J. Med..

[33] Neal B., Perkovic V., Mahaffey K.W., de Zeeuw D., Fulcher G., Erondu N., Shaw W., Law G., Desai M., Matthews D.R. (2017). Canagliflozin and Cardiovascular and Renal Events in Type 2 Diabetes. N. Engl. J. Med..

[34] Zinman B., Wanner C., Lachin J.M., Fitchett D., Bluhmki E., Hantel S., Mattheus M., Devins T., Johansen O.E., Woerle H.J. (2015). Empagliflozin, Cardiovascular Outcomes, and Mortality in Type 2 Diabetes. N. Engl. J. Med..

[35] Perkovic V., Jardine M.J., Neal B., Bompoint S., Heerspink H.J.L., Charytan D.M., Edwards R., Agarwal R., Bakris G., Bull S. (2019). Canagliflozin and Renal Outcomes in Type 2 Diabetes and Nephropathy. N. Engl. J. Med..

[36] McMurray J.J.V., Solomon S.D., Inzucchi S.E., Køber L., Kosiborod M.N., Martinez F.A., Ponikowski P., Sabatine M.S., Anand I.S., Bělohlávek J. (2019). Dapagliflozin in Patients with Heart Failure and Reduced Ejection Fraction. N. Engl. J. Med..

[37] Packer M., Anker S.D., Butler J., Filippatos G., Pocock S.J., Carson P., Januzzi J., Verma S., Tsutsui H., Brueckmann M. (2020). Cardiovascular and Renal Outcomes with Empagliflozin in Heart Failure. N. Engl. J. Med..

